# Perinatal-lethal Gaucher disease presenting as hydrops fetalis

**DOI:** 10.11604/pamj.2015.21.110.7052

**Published:** 2015-06-10

**Authors:** Emira BenHamida, Imene Ayadi, Ines Ouertani, Maroua Chammem, Ahlem Bezzine, Riadh BenTmime, Leila Attia, Ridha Mrad, Zahra Marrakchi

**Affiliations:** 1 Neonatology Department, Charles Nicolle Hospital, Tunis-El Manar University, Tunis, Tunisia; 2 Genetic Department, Charles Nicolle Hospital, Tunis-El Manar University, Tunis, Tunisia; 3 Gynecology Department, Charles Nicolle Hospital, Tunis-El Manar University, Tunis, Tunisia

**Keywords:** Gaucher disease, lysosomal storage disorder, perinatal form, hydrops

## Abstract

Perinatal-lethal Gaucher disease is very rare and is considered a variant of type 2 Gaucher disease that occurs in the neonatal period. The most distinct features of perinatal-lethal Gaucher disease are non-immune hydrops fetalis. Less common signs of the disease are hepatosplenomegaly, ichthyosis and arthrogryposis. We report a case of Gaucher's disease (type 2) diagnosed in a newborn who presented with Hydrops Fetalis.

## Introduction

Gaucher disease (GD), is an autosomal recessive lysosomal storage disorder, caused by a deficiency in the enzyme glucocerebrosidase. It is the most common of the lysosomal storage disorders. Type 2 GD, the most severe and progressive form, manifests either prenatally or in the first months of life [1]. Perinatal-lethal GD is very rare and is considered a variant of type 2 GD. The most distinct features are non-immune hydrops fetalis [2, 3]. We report a case of type 2 Gaucher's disease diagnosed in a newborn who presented with hydrops fetalis.

## Patient and observation

The patient, a female newborn, was the second infant from a 31 year-old gravida 2 para 2 mother. The maternal past medical history included a previous preterm male stillborn with undiagnosed case of non-immune hydrops fetalis. Family history revealed also two undiagnosed cases of hydrops fetalis in two preterm stillbirths. Third trimester fetal ultrasonography revealed severe hydrops fetalis with skin edema, polyhydramnios, hepatomegaly, clustered bowel loops, and fetal hypokinesia. Maternal prenatal tests showed blood type A, Rh +, negative maternal serum indirect Coombs test, immunity to rubella and negative serology results for toxoplasma, hepatitis surface antigen and cytomegalovirus. The infant was delivred by cesarean section indicated for breech presentation and polyhydramnios, at 36 weeks of gestation. Apgar scores of 2 and 3 at 1 and 5 minutes, respectively. Body weight was 2600gm (39^th^ percentile), length was 44 cm (10^th^ percentile), and head circumference was 34 cm (82^th^percentile). At birth, she was apneic and required endotracheal intubation.

**Figure 1:**
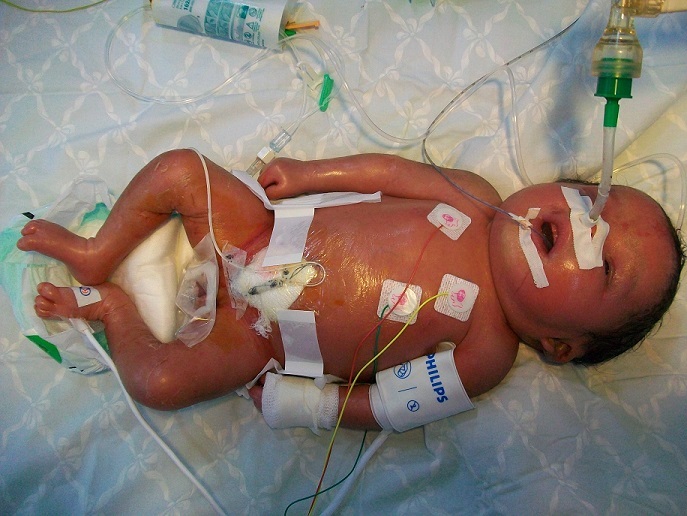
Neonatal presentations of Gaucher disease demonstrating hydrops, peeling and shiny skin

**Figure 2:**
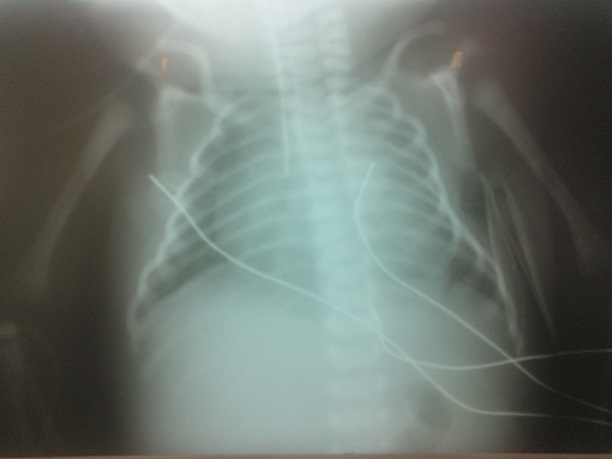
Chest radiograph showing an enlarged cardiac silhouette

Physical examination revealed a state of hydrops with generalized skin edema, thickened and shiny skin, hepatosplenomegaly, hypertonia, arthrogryposis and akinesia. No Dysmorphic facial features or other congenital anomalies were associated (Figure 1). Chest radiograph showed an enlarged cardiac silhouette (Figure 2). Laboratory results included blood type A, Rh +, negative direct Coombs test, a normal blood cell count. Bacterial and viral cultures are negative, SGOT / SGPT: 237/22U/L. Urinary screening for metabolic disorders was negative. Specific tests was as follows: low glucocerebrosidase enzymatic activity on leucocytes (patient level =0.4 nmol/h/mg, controls = 20 nmol/h/mg); and high serum chitotriosidase level (patient = 16 200 nmol/h/ml, controls 200 nmol/h/ml). Parent's measurement of glucocerebrosidase enzymatic activity on leucocytes showed results consistent with a heterozygous for GD. The infant died on the first day of life after stopping resuscitation. Diagnosis was confirmed by direct sequencing of glucose rebrosidase gene (GBA). A single nucleotide substitution was found in exon 9: c.1255G > A leading to the substitution of Aspartic Acid by Asparagine (p.Asp419Asn). This diagnosis has allowed two prenatal diagnoses for the next pregnancies. The foetuses were unaffected.

## Discussion

Hydrops fetalis, has been associated with several lysosomal storage disorders, including Type 2 GD [3]. Cases involving hydrops tend to be perinatal lethal disorders and the pathophysiology is not well understood [4]. Generally, when hydrops is present in Type 2 GD, the fetus dies in utero, or is delivered prematurely and dies within the first days of life [5]. In our case, there were three family histories of undiagnosed perinatal death in the same circunstances. Our patient was intubated and maintainded under mechanical ventilation just to perform laboratory tests and enzymes studies, necessary to inform future pregnancies. When a lysosomal storage disease is suspected in a fetus or a new born because of hydrops fetalis or neonatal hepatosplenomegaly, the presence of arthrogryposis and ichthyosis should raise suspicion for GD. In our case, the infant had a shiny and thickened skin, reminiscient of a collodion-baby phenotype. This finding may have potential diagnostic importance. Patients with Type 2 GD exhibit epidermal abnormalities regardless of whether ichthyosis is clinically evident [5, 6]. The most reliable diagnostic methods are measurement of glucose rebrosidase enzymatic activity and direct sequencing of GBA1. Enzymatic assays can be performed on leukocytes, fibroblasts, or tissue samples such as liver or spleen. Genetic testing for GD by direct sequencing of GBA1 can be used to determine disease and carrier status [7]. The occurrence of an index case invites to offer genetic counselling to parents. Unusual mechanisms including de novo mutations have been described in patients with GD [8]. Thus the recurrence risk is not always obvious. In the presented case, the parents were heterozygous for GD, with a significant risk of recurrence of the disease in their offspring. The diagnosis has allowed genetic counselling in the next pregnancy.

## Conclusion

A greater physician awareness of hydrops fetalis as a possible clinical presentation of GD will allow early recognition and therefore prompt diagnosis, moreover it will allow better estimation of the incidence of this disease. An accurate and timely diagnosis of GD is critical for genetic counselling, patient and family management that can inform future pregnancies.
